# Peritoneal dialysis catheter removal at the time or after kidney transplantation: a systematic review and meta-analysis

**DOI:** 10.1007/s00423-022-02637-y

**Published:** 2022-08-09

**Authors:** Michał Zawistowski, Joanna Nowaczyk, Piotr Domagała

**Affiliations:** 1 Department of General and Transplantation Surgery, Medical University of Warsaw, Nowogrodzka 59, 02-006 Warsaw, Poland; 2 Military Institute of Medicine, Warsaw, Poland

**Keywords:** Peritoneal dialysis catheter, Catheter removal, Kidney transplantation, Delayed graft function

## Abstract

**Purpose:**

An increasing number of patients treated with peritoneal dialysis eventually undergo kidney transplantation. Owing to opposing reports, we aimed to find evidence about the best time for peritoneal dialysis catheter removal in transplant patients.

**Methods:**

We conducted a systematic review and random effects meta-analysis of non-randomized studies of intervention comparing patients with peritoneal dialysis catheters left in place or removed during kidney transplantation in regard to the need for dialysis and occurrence of catheter-related complications. We searched (last update on 8 December 2021) PubMed, Embase, Scopus, and Web of Science for eligible studies. ROBINS-I tool and funnel plot asymmetry analysis were used to assess the quality of included articles.

**Results:**

Eight observational studies were evaluated. Five of them, which involved 338 patients, were included in a meta-analysis. All were at moderate to serious risk of bias. The odds of needing dialysis are more than twice as high for patients with peritoneal dialysis catheters left in situ (pooled odds ratio, 2.21; 95% confidence interval [CI], 1.03 to 4.73; *I*^2^ = 0%). No statistically significant difference was noted when adult and pediatric subgroups were compared (*Q* = 0.13, *P* = .720). More individuals with catheters left in place required dialysis (pooled prevalence, 20.9%; 95% CI, 13.6 to 30.7%; *I*^2^ = 59% vs. 12.4%; 95% CI, 5.6 to 25.2%; *I*^2^ = 0%) and experienced catheter-related infections.

**Conclusion:**

Available evidence is scarce. Unless new data from a randomized controlled trial are available, the dilemma of peritoneal dialysis catheter removal cannot be solved.

**Trial registration:**

PROSPERO Protocol ID: CRD42020207707.

**Graphical abstract:**

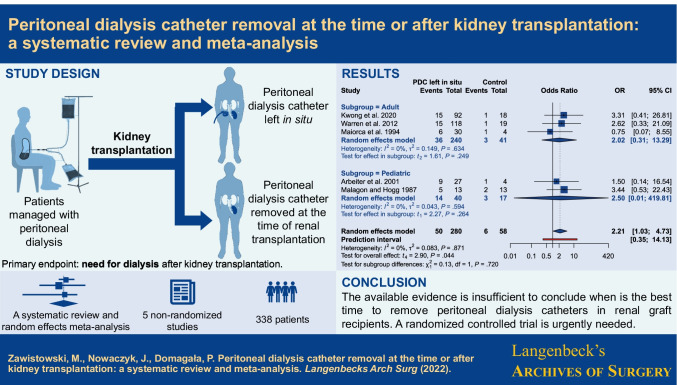

## Introduction

Peritoneal dialysis is a renal replacement therapy that is often preferred by patients with end-stage renal disease as it usually has less impact on their daily routine than hemodialysis [1]. Peritoneal dialysis is also favored in the pediatric population. Although in many countries the peritoneal dialysis-first strategy is often a default policy nowadays, kidney transplantation is still considered a superior method that usually guarantees the best possible quality of life [2]. Therefore, an increasing number of patients undergoing peritoneal dialysis eventually receive their first renal graft. An ongoing debate concerns the management of peritoneal dialysis catheters during transplantation [3]. One of the questions that need to be answered is whether the catheter should be removed at the time or after the transplant procedure.

Current policies differ in many transplant centers. Practices range from routine catheter removal simultaneously with transplantation [4, 5] to postponing it until good renal function is achieved or until a particular postoperative time [6, 7]. The problem is mentioned briefly in the European Best Practice Guidelines (EBPG) for Peritoneal Dialysis from 2005 [8]. Over a decade ago, the EBPG Expert Group on Peritoneal Dialysis stated that leaving peritoneal dialysis catheters in situ for 3–4 months is acceptable even with a functioning renal graft, although quicker removal was advised if possible. However, evidence back then was poor and the methodology used to create these guidelines is not considered robust [9, 10].

To solve the dilemma of peritoneal dialysis catheter removal, several aspects should be considered. The first one is the choice of a proper measure to evaluate both interventions. The main argument for leaving catheters in place is having dialysis access ready to use in case of delayed graft function or graft failure [11]. Therefore, we believe that there is a need to compare the overall need for dialysis early after transplantation between the two groups of patients, with catheters left in situ and removed simultaneously with transplantation. Furthermore, the used dialysis modality should be evaluated to confirm the feasibility of peritoneal dialysis in such cases. Additionally, complications associated with each procedure should be analyzed. Finally, such factors as patients’ quality of life and costs should be considered.

We aimed to evaluate available evidence about the peritransplant management of peritoneal dialysis catheters using a critical and systematic approach. We focused on the timing of their removal. A systematic review and random effects meta-analysis of non-randomized studies of intervention was conducted.

## Materials and methods

### Reporting guidelines and protocol registration

The study results are reported in compliance with the Preferred Reporting Items for Systematic Reviews and Meta-Analysis (PRISMA) Statement and the Meta-analysis Of Observational Studies in Epidemiology (MOOSE) guidelines [12, 13]. Our study conforms to the recommendations of the Study Center of the German Society of Surgery [14]. We followed a prospectively written protocol that is summarized and recorded in the PROSPERO database (CRD42020207707).

### Inclusion and exclusion criteria

Articles were included in the systematic review if they compared or reported outcomes of leaving peritoneal dialysis catheters in situ with removing them at the time of surgery. Full-text peer-reviewed papers, as well as conference abstracts of acceptable quality, were evaluated. The exclusion was based on the following criteria: animal studies, no control group, non-English, retracted articles, reviews, editorials, and case reports or case series. Studies with numbers of patients needing post-transplant dialysis reported for both groups were considered for inclusion in a meta-analysis.

### Studied groups and outcomes

The experimental group consisted of patients with the peritoneal dialysis catheter left in situ, whereas in the control group it was removed at the time of surgery. Additionally, adult and pediatric subgroups were distinguished. The main outcome was defined as the need for dialysis in the early period after transplantation — that is not later than within the first two postoperative months. Secondary endpoints included catheter-related complications (peritonitis, exit-site, inner cuff, and tunnel infections). In Table 1, we summarized the PICO (Population, Intervention, Comparison, Outcome) framework for this study.

**Table 1:** The PICO framework

Population	Adult and pediatric patients undergoing renal transplantation after being maintained on peritoneal dialysis
Intervention	Leaving the peritoneal dialysis catheter in situ during kidney transplantation
Comparison	Removing the peritoneal dialysis catheter at the time of kidney transplantation
Outcome	The need for dialysis in the early period after transplantation (not later than within the first two postoperative months), catheter-related complications (peritonitis, exit-site, inner cuff, and tunnel infections)

### Literature search

We searched (last update on 8 December 2021) PubMed, Embase, Scopus, and Web of Science databases for studies eligible for further investigation. The search strategy is reported in Table 2. No dates of coverage nor language restrictions were applied at this stage. Search queries were reviewed with the PRESS Peer Review Checklist [15]. All retrieved citations were exported, deduplicated, and processed using the Zotero 5.0.89 software (Center for History and New Media, Fairfax, Virginia, USA) [16]. Moreover, reference sections of evaluated papers were manually searched for additional records.

**Table 2:** Search strategy

Database	Date of search (repeated search)	Search query	Search filters	Number of found publications (repeated search)
PubMed	9/5/2020 (12/08/2021)	((Peritoneal Dialysis[MeSH Terms] AND (Catheters[MeSH Terms] OR Catheters, Indwelling[MeSH Terms])) OR “PD catheter”) AND remov*	Human	491 (528)
Embase	9/5/2020 (12/08/2021)	'peritoneal dialysis catheter'/exp AND 'catheter removal'/exp	-	190 (234)
Scopus	9/5/2020 (12/08/2021)	TITLE-ABS-KEY ( ( peritoneal AND dialysis AND catheter) AND ( catheter AND remov*) AND ( ( kidney OR renal) transplant*))	-	216 (236)
Web of Science	9/5/2020 (12/08/2021)	(peritoneal dialysis catheter) AND (catheter remov*) AND ((kidney OR renal) transplant*)	Search for “All Fields”	144 (168)
Number of all found articles				1041 (1166)
Number of all found articles without duplications				825 (915)

### Data extraction

The search results were independently analyzed by two investigators. Discrepancies were resolved by discussion and consensus among all co-authors. The researchers extracted the following information: study design, publication year, a place where it was conducted, number of participants in each group, population studied (adult/pediatric), criteria for peritoneal dialysis catheter removal, number of events of needing post-transplant dialysis with details, and prevalence or ratios of surgical and infectious complications (including peritonitis and other catheter-related infections).

In addition, corresponding authors of two studies were contacted to obtain some missing data. Unfortunately, no response was received after making three attempts in each case.

### Quality assessment

The risk of bias for observational studies was assessed using the ROBINS-I tool [17]. This method is recommended by the Cochrane Scientific Committee when non-randomized designs are applied for the investigation of interventions. Furthermore, funnel plots were visually checked for asymmetry that might indicate publication bias. The use of the Egger test would only be justified if at least ten studies were included in the meta-analysis [18]. A GRADE evidence profile was created in the GRADEpro GDT software (https://gradepro.org/) to rate the quality of evidence regarding the primary endpoint [19, 20].

### Statistical analysis

For studies with the primary event data reported, unadjusted odds ratios with 95% confidence intervals were calculated and pooled using a random effects model and the Hartung-Knapp-Sidik-Jonkman adjustment [21]. The between-study heterogeneity was evaluated with *I*^2^ statistics. A prediction interval was also calculated to estimate the true effect size if a new study was performed. Subgroup analysis was conducted to identify potential differences between the pediatric and adult populations. Meta-analysis of proportions with a random effects model was used to calculate the pooled prevalence of needing dialysis in each group with 95% confidence intervals. Additionally, frequencies of episodes of peritonitis and other catheter-related infections were estimated with this method. All analyses were completed using the meta [22], the matafor [23], the PRISMA2020 [24], and the robvis [25] packages in R 4.2.0 statistical environment (R Core Team, 2022) [26].

## Results

### Study selection and characteristics

**Fig. 1:**
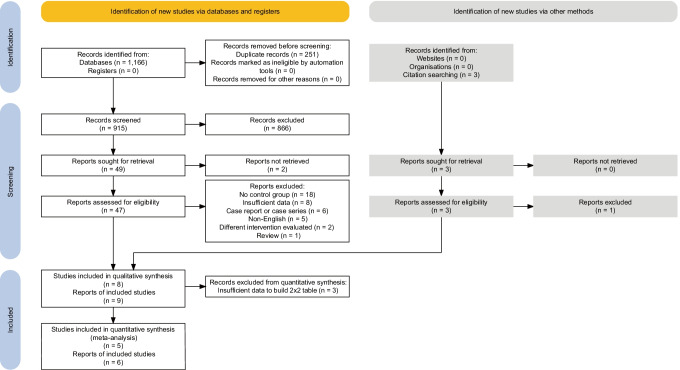
PRISMA 2020 flow chart of the study screening and selection for the systematic review

We identified 915 non-duplicate records that were independently screened by two researchers. The selection process is shown in Fig. 1. No prospective studies were found. All articles included in this qualitative and quantitative synthesis were of retrospective cohort design. Forty-five percent (18/40) of papers were excluded due to the absence of a control group. It was either not present by design or results of comparators were simply not reported. Eight studies described in nine papers (one was a conference abstract by Warren et al. which was subsequently published as a full-text article [27]) were included in the final systematic review. Results from five of them could be synthesized in a meta-analysis. A total of 338 patients were evaluated by these publications. Their characteristics and main findings are summarized in Table 3.

**Table 3:** Studies included in the systematic review, their characteristics, and reported results

Study	Country	Number of living/deceased donor transplants	Mean/median age of patients, yr (SD)/[range]	Mean/median time to PDC removal, mo (SD)/[range]	Prevalence of peritonitis in the “in situ” group, % (*n*/*N*)	Prevalence of catheter-related infections in the “in situ” group, % (*n*/*N*)	Prevalence of catheter-related infections in the control group, % (*n*/*N*)	Successful use of the pretransplant PDC when dialysis was needed, % (*n*/*N*)
Adult patients								
Kwong et al. [11]	Canada	33/77	53.1 (13)^a^	N/A	1.1 (1/92)	N/A	0	80 (12/15)
Warren et al. [27]	Canada/UK	N/A	N/A	N/A	8.5 (10/118)	10.2 (12/118)	0	80 (12/15)^b^
Maiorca et al. [32]	Italy	0/34	39 (10)	17 (8) d	3.3 (1/30)	N/A	0	100 (6/6)
O'Donoghue et al. [33]	UK	0/241	Mean of 37.1	[0–371]	3.8 (9/234)	6.4 (15/234)	14.3 (1/7)	N/A
Pediatric patients								
Arbeiter et al. [34]	Austria	2/29	Mean of 6.8 [1–15]	Median of 3	11.1 (3/27)	18.5 (5/27)	0	N/A
Andreetta et al. [31]	Italy	9/71	Mean of 9.3 [1.7–21]	Mean of 80.3 [0–216] d	0^c^	0^c^	0^c^	100^d^ (12/12)
Malagon and Hogg [30]	USA	Experimental group: 0/13; control group: 13/0	9 (4.2) [1–15]	Mean of 3.8 weeks [10 days–5 weeks]	7.7 (1/13)	15.4 (2/13)	7.7 (1/13)	N/A
Schärer and Fine [54]	Various countries^e^	N/A	All < 15	22 [2–113] d	9.4 (9/96)	N/A	N/A	N/A

*Abbreviations*: *N/A*, not available; *PDC*, peritoneal dialysis catheters; *SD*, standard deviation

^a^Calculated also for patients not receiving PD but reported in the study

^b^Five patients developed peritonitis, 3 others needed the conversion to hemodialysis due to dialysate-derived fluid leaks

^c^Six patients were lost to follow-up and their data were not provided — concern about the presence of bias due to missing data. Furthermore, 1 peritonitis case due to sepsis was not classified as catheter-related

^d^Additionally, 2 children restarted peritoneal dialysis at 4 and 12 months after surgery. Their pretransplant catheters were successfully used

^e^It was a questionnaire-based cooperative study involving respondents from the USA, Germany, Denmark, Canada, France, Israel, Finland, Switzerland, UK, Japan, Sweden, and the Netherlands

### Risk of bias and study quality

**Fig. 2:**
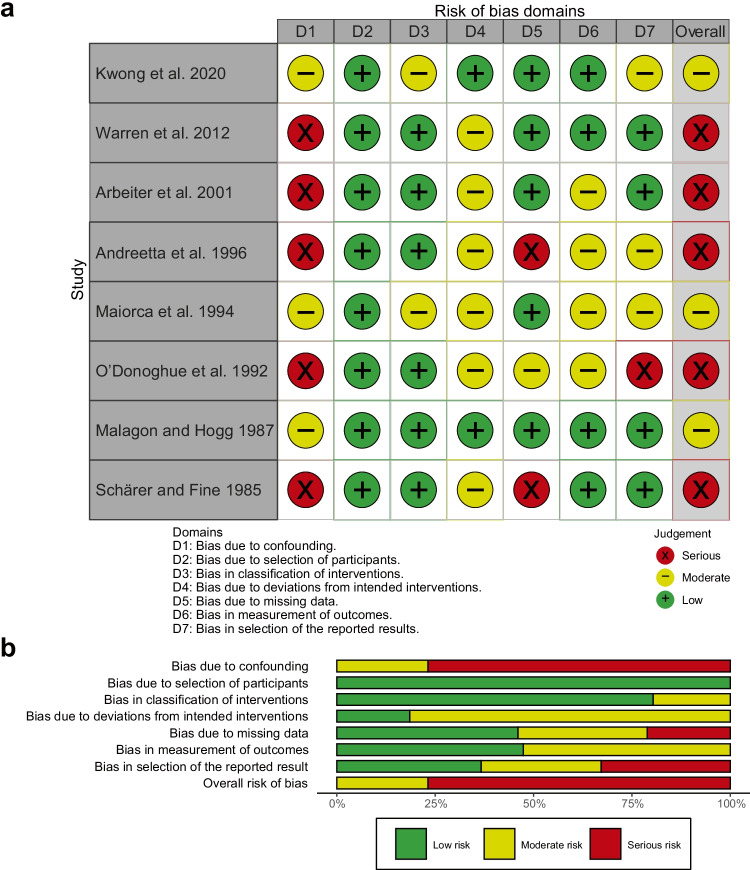
“Traffic light” (**a**) and weighted bar (**b**) risk of bias charts for studies included in the systematic review

**Fig. 3:**
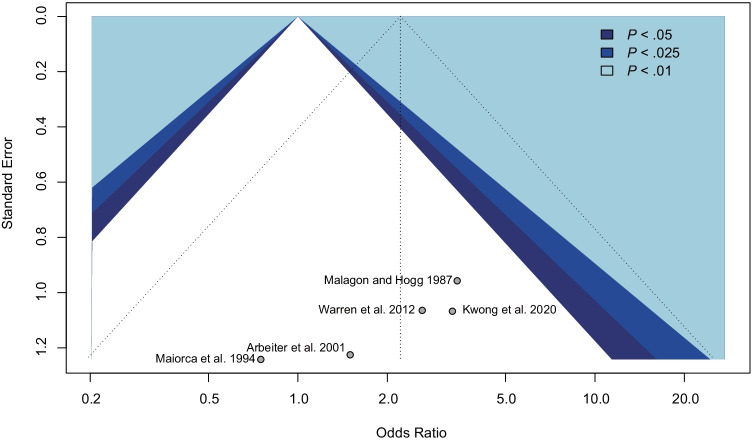
Funnel plot for the assessment of publication bias

We used the ROBINS-I tool to assess the risk of bias in seven domains (Fig. 2). The reports were of moderate or low quality. Furthermore, a funnel plot was created (Fig. 3) but no formal test for its asymmetry was performed due to the inclusion of fewer than 10 studies. Therefore, publication bias cannot be excluded. Low heterogeneity (*I*^2^ < 25%) is present in the primary analysis regardless of the subgroup studied. The numbers of participants are small and insufficient in all papers to guarantee satisfactory power of the used statistical tests. All studies are with level 4 evidence according to the 2011 Oxford Centre for Evidence-Based Medicine Levels of Evidence [28].

### The GRADE evidence profile

In Table 4, we report the GRADE evidence profile with explanations. Due to the presence of serious risk of bias, indirectness, very serious imprecision, and suspected existence of publication bias, the final certainty of evidence regarding the primary outcome was graded as very low [20, 29].

**Table 4:** The GRADE evidence profile

Certainty assessment							№ of patients		Effect		Certainty	Importance
№ of studies	Study design	Risk of bias	Inconsistency	Indirectness	Imprecision	Other considerations	PDC left in situ	Control	Relative (95% CI)	Absolute (95% CI)		
The need for dialysis in the early period after transplantation (not later than within the first two postoperative months)												
5	Observational studies	Serious^a^	Not serious^b^	Serious^c^	Very serious^d^	Publication bias strongly suspected^e^	50/280 (17.9%)	6/58 (10.3%)	OR 2.21 (1.03 to 4.73)	100 more per 1000 (from 3 to 250 more)	⨁◯◯◯ Very low	Important

*Abbreviations*: *CI*, confidence interval; *OR*, odds ratio

^a^Based on the ROBINS-I tool assessment

^b^Low heterogeneity (*I*^2^ < 25%) was present regardless of the subgroup studied

^c^Due to differences between study populations and in outcome measures

^d^Fewer than 300 patients for each outcome

^e^Publication bias cannot be excluded based on the visual inspection of funnel plot asymmetry

### Peritransplant peritoneal dialysis management protocols

There are various standards adopted by transplantation centers regarding the management of patients undergoing renal transplantation after being maintained on peritoneal dialysis. Nevertheless, most authors agree that peritoneal dialysis catheters might be routinely removed at the time of surgery in living renal donor recipients due to a significantly lower risk of delayed graft function [11, 30–32]. In some studies, suspicion of a catheter-related (e.g., exit-site or tunnel) infection was classified as another indication for concurrent catheter removal [33–35]. Contrastingly, in some centers, such patients were temporarily excluded from the waiting list until recovery [30, 32]. A similar approach was implemented by most authors in regard to the presence of peritonitis. Safe waiting time from the recent incidence of peritonitis to transplantation was specified as between 1 and 3 weeks [32, 33].

The time to peritoneal dialysis catheter removal after successful transplantation was set at 3 months by Arbeiter et al. [34] and 8 to 12 weeks by O'Donoghue et al. [33]. Malagon and Hogg [30] did this before patients’ discharge when stable graft function was confirmed. The actual interval times reported in these papers are summarized in Table 3.

### Need for dialysis in the early period after transplantation

**Fig. 4:**
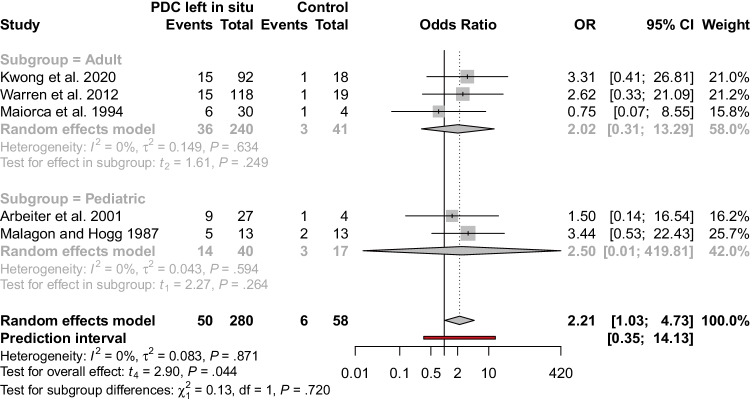
Forest plot with the subgroup analysis. Non-adjusted odds ratios for the need of dialysis in patients with PD catheter left in situ (the experimental group) and those after its removal at the time of kidney transplantation (the control group). *PDC*, peritoneal dialysis catheter

The main outcome that was analyzed in our study was the use of dialysis (any modality) up to 2 months after transplantation. By combining the data by meta-analysis with a random effects model, we calculated that odds of needing dialysis are more than twice as high for patients with the catheter left in situ (pooled unadjusted odds ratio, 2.21; 95% confidence interval, 1.03 to 4.73). However, given the prediction interval of 0.35 to 14.13, it is possible that in some future studies, the odds might as well be higher in the control group. None of the evaluated papers reported adjusted odds ratios. We did not find differences in the overall effect between the adult and pediatric subgroups (*Q* = 0.13, *P* = 0.720). The results of this analysis are presented in a forest plot (Fig. 4).

**Fig. 5:**
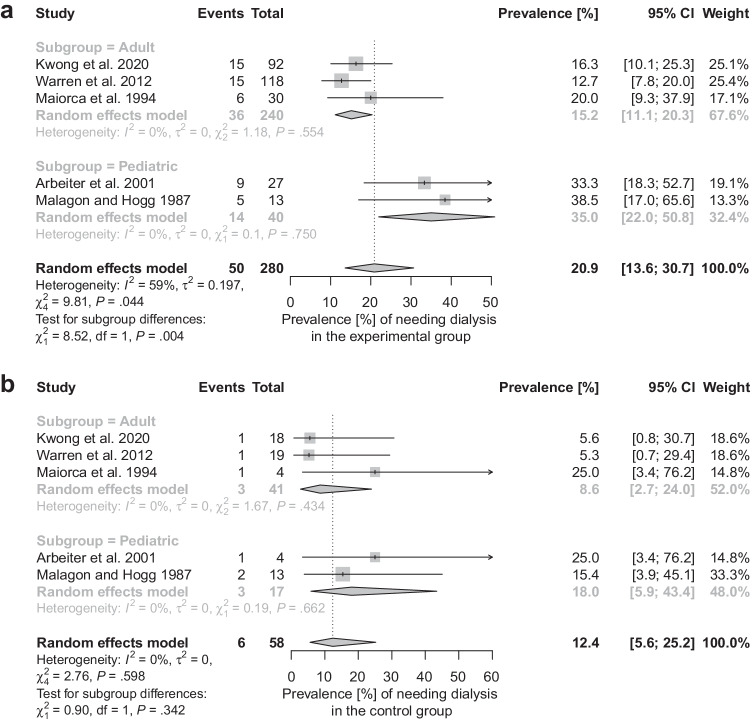
Forest plots showing the pooled prevalence of needing dialysis by patients in the experimental (**a**) and control (**b**) groups

The prevalence of needing dialysis (within the first two postoperative months) in the experimental group was 20.9%; 95% confidence interval, 13.6 to 30.7% while for those with the catheter removed, it was 12.4%; 95% confidence interval, 5.6 to 25.2% (Fig. 5). There was a noticeable difference between the pediatric and adult populations in the group with peritoneal dialysis catheters left in place (*Q* = 8.52, *P* = 0.004; Fig. 5a).

When dialysis was necessary in the group with patients from the “in situ” group, the peritoneal dialysis catheter utilization was feasible in most cases (Table 3). However, this information was reported only in half of the evaluated papers.

### Prevalence of peritonitis and other catheter-related infections

**Fig. 6:**
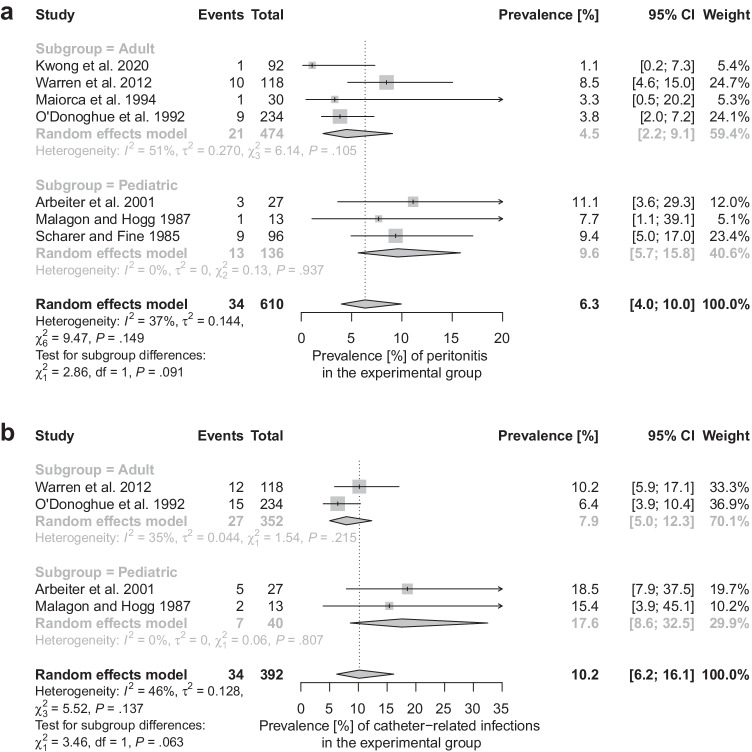
Forest plots showing the pooled prevalence of peritonitis (**a**) and catheter-related infections (**b**) in patients with peritoneal dialysis catheters left in place after kidney transplantation

Pooled incidence of peritonitis in patients whose catheters were left in situ is 6.3%; 95% confidence interval, 4.0 to 10.0% (a study by Andreetta et al. [31] was excluded from this calculation owing to concern of bias due to missing data, Fig. 6a). For all catheter-related infections (including peritonitis as well as exit-site or tunnel infections), the pooled frequency was 10.2%; 95% confidence interval, 6.2 to 16.1% (Fig. 6b). These complications were rare in individuals with the catheter removed at the time of transplantation. O'Donoghue et al. [33] with Malagon and Hogg [30] described only single such cases (Table 3).

## Discussion

In this systematic review, we summarized the current evidence on the peritransplant management of patients with peritoneal dialysis catheters. The quality of available data is poor and the clinical question of whether peritoneal dialysis catheters should be removed at the time of surgery or not cannot be definitely answered. However, experience emerging from the published articles might be useful for clinicians and patients in the decision-making process.

The lack of evidence-based guidelines leads to the presence of varying policies adopted by different centers and specialists. Some routinely leave the catheters in situ [4, 5, 36–40] while others remove them as a rule during the transplant procedure [6, 7]. There are certain situations when indwelling peritoneal catheters should be removed before or at the time of kidney implantation, including infectious (peritonitis, exit-site/tunnel/inner cuff infection), non-infectious and mechanical (broken catheter, catheter migration, pericatheter leak, flow dysfunction, peritoneal loss of function, peritoneal membrane breach, pleuri-peritoneal communication, sclerosant peritonitis), and some rare complications (allergic reaction, organ erosion, severe infusion/pressure pain, hemoperitoneum) [41, 42]. Additionally, some authors suggest that their immediate removal ought to be considered in living donor procedures that are associated with a lower risk of delayed graft function [3, 11, 30, 31]. Some protocols of the evaluated studies had already implemented that as a rule, which resulted in the different prevalence of living donor recipients in the experimental and the control groups. We suspect that it may be a confounding factor, but its true effect could not be formally determined. Maiorca et al. [32] analyzed only deceased donor transplants. It is the only study with odds of needing dialysis lower for patients with peritoneal dialysis catheters left in situ (Fig. 4). However, the control group consisted of only four patients. Moreover, Malagon and Hogg [30] described removing the catheter only in living-donor kidney recipients. None of them had delayed graft function but two developed surgical complications which finally led to the need for dialysis. Other identified indications for simultaneous catheter removal included concurrent renal and pancreas transplantation as well as having peritoneum compromised for other reasons [3, 11, 43].

It is believed that leaving a peritoneal catheter in situ can be considered when the probability of its subsequent use is substantial. It applies mainly to deceased donor transplant recipients in whom the prevalence of delayed graft function is between 10 and 30% [44]. The risk is even higher for organs from extended criteria donors [35]. However, there is no consensus on how long the removal should be postponed (some studies evaluated by us report that peritoneal dialysis catheters were used mostly within the first post-transplant month [30, 34, 40]). Extended time is associated with more complications, mainly infectious, that in this group are particularly dangerous owing to immunosuppressive therapy [7, 27, 34, 45]. In our meta-analysis, we calculated that pooled prevalence of catheter-related infections in patients with Tenckhoff catheters left intact was 10.2%; 95% confidence interval, 6.2 to 16.1% (Fig. 6b). Owing to data incompleteness, we could not evaluate the correlation between the time for which catheters were left in place and the incidence of catheter-related infections. Some authors still argue that in many cases these conditions (peritonitis, exit-site, and tunnel infections) can be managed with antibiotics and a sensible number of patients without complications still benefit from resuming peritoneal dialysis in case of early graft failure [33, 46]. However, no objective (medical and economic) data are available to support this opinion.

In a recent commentary on a case–control study by Gardezi et al. [47], Issa and Lakhani [48] proposed an algorithm for the management of peritoneal dialysis catheter in patients undergoing renal transplantation. These experts suggest leaving peritoneal dialysis catheters in place only when (1) delayed graft function is highly expected and (2) peritoneum breach as well as catheter-related infection is absent. Otherwise, it should be removed at the time of transplant or before hospital discharge. Nevertheless, the latter exposes the patient to the risk of additional surgical procedures. Moreover, the methods to predict delayed graft function in individual patients are still a matter of debate. Issa and Lakhani propose the use of Kidney Donor Profile Index (KDPI) along with several other predictive factors (such as long cold ischemia time and recipient’s history of obesity, diabetes mellitus, prior allosensitization, long dialysis vintage, and waiting time) [48]. However, predictive models for delayed graft function are not well clinically validated. Furthermore, KDPI was not meant and developed to predict delayed graft function. The available evidence shows that these two factors are not even well correlated [49, 50]. The discussed algorithm should be precisely investigated in a prospective randomized clinical trial.

Using random effects meta-analysis, we calculated that patients requiring post-transplant dialysis more likely had their dialysis catheters left intact during transplantation than those not needing it (pooled unadjusted odds ratio, 2.21; 95% confidence interval, 1.03 to 4.73, Fig. 4). This finding suggests that protocols followed by the centers, from which the data were obtained, are effective to a certain extent. More individuals with peritoneal dialysis catheters left in situ needed dialysis (pooled prevalence, 20.9%; 95% confidence interval, 13.6 to 30.7% vs. 12.4%; 95% confidence interval, 5.6 to 25.2%, Fig. 5), as expected.

When dialysis was needed early after transplantation and a peritoneal dialysis catheter had not been removed at the time of surgery, some studies report that in up to 80–100% of cases peritoneal dialysis was feasible [11, 27, 31, 32]. However, in the group studied by Warren et al. [27], in five of such patients, the peritonitis occurred, and three others had to be converted to hemodialysis. In a report by Rizzi et al. [51] who analyzed 313 patients whose catheters were left during transplantation, almost 16% of them required dialysis. Among them, only 33% could benefit from peritoneal dialysis access being available — others were referred to hemodialysis. Taking into account that only for a few studies are these data known, the high prevalence of these catheters’ utilization might be overestimated. In one study (excluded from the final analysis due to lack of a control group), patients requiring dialysis right after transplantation were electively hemodialyzed even with a peritoneal dialysis catheter left in situ [35]. Only those with primary non-function were transferred to peritoneal dialysis immediately (2.5%, 3/120). Such an approach stands against the fact that peritoneal dialysis catheters might be safely used early after renal transplantation [45, 52].

Patients whose peritoneal catheters had been removed at the time of transplantation might also require dialysis. They are usually treated with hemodialysis which could possibly lead to some complications. However, no undesired events were reported in the analyzed studies [11, 27]. Furthermore, McGregor [3] pointed out that many patients undergoing renal transplantations have a central line placed by an anesthesiologist. In such cases, it is relatively simple and safe to replace it with a dialysis line when necessary. Some individuals may as well be managed with peritoneal dialysis. Malagon and Hogg [30] described two living-donor graft recipients who needed dialysis because of surgical complications (kidney laceration in one case and vascular damage in the second). They had new peritoneal dialysis catheters placed during their grafts’ repair surgeries and the therapy was uneventful.

The discussion about the timing of indwelling peritoneal catheter removal involves consideration of some costs and benefits. Most patients with such dialysis access left in situ during transplantation eventually require a second intervention to take it out. There are various complications linked to the presence of a foreign body in the abdomen. It is not always feasible to perform peritoneal dialysis even with the Tenckhoff or similar catheter available, as discussed above. The benefits are avoiding potential problems associated with vascular access for hemodialysis, the possibility of quick resumption to peritoneal dialysis in case of temporal or permanent graft failure, and having a route ready to drain potential ascites. However, owing to the unpleasant experience with catheter-related complications in renal graft recipients with catheters left in place, some authors declared to switch to a routine of removing them at the time of transplantation [3, 27]. As the certainty of the available is very low, we believe that ultrasound examination should be considered in select uncertain cases [53].

### Limitations

This study is limited by the lack of high-quality evidence. Our synthesis was based on retrospective imbalanced studies of moderate or low quality. We summarized their findings in a meta-analysis including only 338 patients. We were only able to obtain odds ratios that were not adjusted for any of the previously identified candidate confounders. We also found relatively old articles which might not well represent current standards of care. Our report proves that further evidence is urgently needed to answer the clinical dilemma of peritransplant peritoneal dialysis catheter management. The medical community might benefit from our experience when designing future trials. As we demonstrated, there are numerous potential confounders to be controlled for when conducting retrospective studies about this problem. Taking into account that 45% of the excluded papers were lacking a control group, it is important to avoid this approach, if not justified. However, it is highly desirable in this case to design and conduct high-quality and adequately powered randomized controlled trial in both the adult and pediatric populations.

## Conclusion

In conclusion, the evidence supporting the choice to remove or to leave peritoneal dialysis catheters in situ during renal transplantation is very weak. Unless supportive data are available, preferably from a randomized controlled trial, the dilemma of peritoneal dialysis catheter removal remains unsolved.
